# Approach to the Pediatric Patient With Glucocorticoid-Induced Osteoporosis

**DOI:** 10.1210/clinem/dgae507

**Published:** 2024-08-10

**Authors:** Leanne M Ward, Sarah A Bakhamis, Khaldoun Koujok

**Affiliations:** Department of Pediatrics, Faculty of Medicine, University of Ottawa and Division of Endocrinology, Children's Hospital of Eastern Ontario, Ottawa, Ontario, Canada, K1H 8L1; Department of Pediatrics, Faculty of Medicine, University of Ottawa and Division of Endocrinology, Children's Hospital of Eastern Ontario, Ottawa, Ontario, Canada, K1H 8L1; Department of Medical Imaging, Faculty of Medicine, University of Ottawa and Division of Pediatric Radiology, Children's Hospital of Eastern Ontario, Ottawa, Ontario, Canada, K1H 8L1

**Keywords:** glucocorticoids, children, osteoporosis, fractures, bone mineral density, treatment

## Abstract

Glucocorticoid (GC) therapy remains the cornerstone of treatment for many conditions of childhood and an important cause of skeletal and endocrine morbidity. Here, we discuss cases that bring to life the most important concepts in the management of pediatric GC-induced osteoporosis (pGIO). Given the wide variety of underlying conditions linked to pGIO, we focus on the fundamental clinical–biological principles that provide a blueprint for management in any clinical context. In so doing, we underscore the importance of longitudinal vertebral fracture phenotyping, how knowledge about the timing and risk of fractures influences monitoring, the role of bone mineral density in pGIO assessments, and the impact of growth-mediated “vertebral body reshaping” after spine fractures on the therapeutic approach. Overall, pGIO management is predicated upon early identification of fractures (including vertebral) in those at risk, and timely intervention when there is limited potential for spontaneous recovery. Even a single, low-trauma long bone or vertebral fracture can signal an osteoporotic event in an at-risk child. The most widely used treatments for pediatric osteoporosis, intravenous bisphosphonates, are currently recommended first-line for the treatment of pGIO. It is recognized, however, that even early identification of bone fragility, combined with timely introduction of the most potent bisphosphonate therapies, may not completely prevent osteoporosis progression in all contexts. Therefore, prevention of first-ever fractures in the highest-risk settings is on the horizon, where there is also a need to move beyond antiresorptives to the study of anabolic agents.

Glucorticoid-induced osteoporosis (GIO) is a potentially debilitating complication of glucocorticoid (GC) therapy in children with myriad underlying disorders that span inflammatory conditions, neuromuscular diseases, malignancies, major organ dysfunction, and organ transplantation. Importantly, the underlying conditions themselves often pose significant threats to bone strength that are amplified by supraphysiologic GC therapy. In such cases, whether the child develops osteoporosis teeters on the balance between effective treatment of the underlying disease and the osteotoxicity of the very therapy used to treat the condition. In some cases, such as pediatric leukemia, effective treatment of the underlying disease with (intermittent) GC therapy usually wins the war. In others, the osteotoxicity of GC therapy may limit the benefits of disease-targeted treatment; for instance, GC-associated fractures causing premature loss of ambulation in Duchenne muscular dystrophy (DMD).

To better understand the natural history of osteoporotic fractures and risk factors in the pediatric GIO (pGIO) setting, the Canadian STeroid-induced Osteoporosis in the Pediatric Population (STOPP) Consortium carried out a 6-year, multicenter observational cohort study in over 400 GC-treated children with various underlying conditions (1-7). Among the most informative observations arising from the STOPP study were that vertebral fractures are a clinical signature of pGIO, they align with the period of maximal GC exposure (and thereby usually occur early in the GC treatment course), and they are frequently asymptomatic (thereby going undetected in the absence of routine spine imaging). The STOPP Consortium also unveiled that even asymptomatic vertebral fractures are associated with an increased risk of future vertebral fractures and, when left untreated, may progress to more advanced collapse in those with persistent risk factors. These observations led to a paradigm shift in the monitoring and diagnosis of GC-treated children, one that placed vertebral fracture identification at the fulcrum of the pGIO assessment. At the same time, children have the growth-mediated ability to restore normal vertebral body dimensions following vertebral fractures, an important index of recovery that may preclude the need for osteoporosis therapy (8).

To bring these key concepts to life, we discuss 3 GC-treated children with underlying diseases that themselves can adversely affect bone strength. In so doing, we highlight the spectacular potential for growth-mediated vertebral body reshaping following vertebral fractures, both with and without bisphosphonate therapy. We also showcase the attenuated response to intravenous (IV) bisphosphonate therapy in a patient with an aggressive, prolonged GC regimen and progressive disease–related risk factors for bone fragility. These cases provide insight into the day to day evaluations and decision-making undertaken by the practitioner and underscore the unmet needs that drive future research in pGIO.

## Illustrative Cases

### Patient 1: 17-Month-Old Girl With Systemic Juvenile Idiopathic Arthritis

A 17-month-old girl with systemic juvenile idiopathic arthritis (sJIA) presented to the Pediatric Bone Health-Osteology Clinic for a routine skeletal health assessment following a 9-month history of GC therapy. She was diagnosed with sJIA at 7 months of age following fever, decreased appetite, lethargy, rash, and swelling of the hands, wrists, and feet. At 7 months of age, she was started on prednisone 1 mg/kg/day (79 mg/m²/day in hydrocortisone equivalents [HCE]), with the peak dose reached at 10 months of age (prednisone 2 mg/kg/day, 166 mg/m²/day in HCE). She was also treated with indomethacin and methotrexate soon after diagnosis. At the time of her bone health assessment, there was no history of fractures or unexplained irritability to suggest bone pain, her motor milestones were on target, and she did not have any functional limitations. Her dietary intake of calcium was adequate for age, and she was adherent to vitamin D_3_ 400 IU/day. At 13 months of age, she started a progressive prednisone wean that was enabled by the prescription of a biological agent (Anakinra, an interleukin-1 receptor antagonist) at 12 months of age, with continuation of the methotrexate.

**Figure 1.**
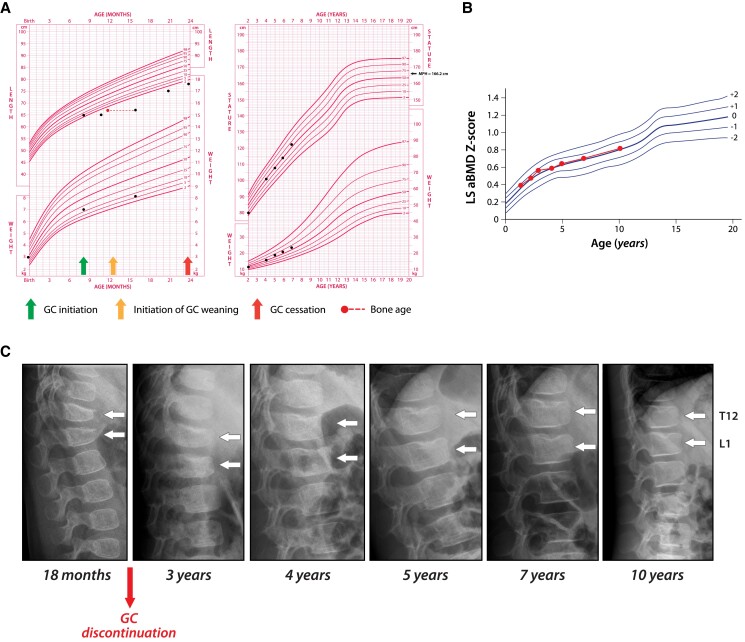
Patient 1. (A-C) Osteoporotic vertebral fractures in a young girl with GC-treated systemic juvenile idiopathic arthritis who underwent complete vertebral body reshaping with GC weaning, well-controlled underlying disease, and significant residual growth potential. (A) highlights growth deceleration while on GC therapy, and an increase in height velocity during and following her GC wean. The increase in height velocity provided an early sign of her potential to undergo spontaneous (medication-unassisted) vertebral body reshaping, informing the decision to withhold osteoporosis-targeted therapy. (B) shows lack of significant impact of GC therapy on the LS aBMD Z-score trajectory. This observation highlights the importance of vertebral fracture identification independent of a given LS aBMD Z-score threshold in the pGIO setting (for further discussion see text). (C) demonstrates this patient's reclamation of near-normal vertebral dimensions following Grade 2, GC-induced vertebral fractures at T12 and L1. She also demonstrated intervertebral disk herniation at the superior endplates of the affected vertebrae (similar to the appearance of Schmorl's nodes). Her young age, significant residual growth potential and resolution of osteoporosis risk factors were the main factors in her improved vertebral body structure. Abbreviations: a, areal; BMD, bone mineral density; GC, glucocorticoid; pGIO, pediatric glucocorticoid-induced osteoporosis; L, lumbar; LS, lumbar spine; MPH, midparental height; T, thoracic.

On physical examination, she had a Cushingoid facies and dorsal fat pad. Her growth chart was consistent with chronic GC exposure, including linear growth deceleration and excess weight for height (Fig. 1A). She had mild hyperkyphosis, absence of joint swelling or rash, lack of long bone deformity, and a normal gait. Biochemical parameters of bone and mineral ion metabolism were normal, including an adequate 25-hydroxyvitamin (25OHD) concentration (defined as >50 nmol/L or 20 ng/dL). At a chronological age of 16 months, her phalangeal bone age (BA) was 12 months and carpal BA 9 months, according to the Atlas of Greulich and Pyle (9). Her lumbar spine (LS) areal bone mineral density (aBMD) Z-score was +0.2, which was within the “normal range” and robust for her height Z-score of −3.6 (Fig. 1B). A routine lateral spine radiograph showed Genant Grade 2 (moderate) vertebral fractures at T12 and L1 (Spinal Deformity Index [SDI] = 4, Fig. 1C), with an exaggerated thoracolumbar kyphosis and loss of lumbar lordosis. Her asymptomatic vertebral fractures underscored the importance of vertebral fracture phenotyping in at-risk children, including the precedence that lateral spine imaging takes over BMD by dual-energy x-ray absorptiometry (DXA) (since the LS BMD Z-score was not low). This is particularly true for very young infants and toddlers, where the skill needed to acquire adequate DXA images may not always be available in a given institution. Given her young age, planned GC wean, and early signs of linear growth recovery from GC excess (Fig. 1A), the decision was made to monitor her skeletal health trajectory optimistically instead of initiating bone protection therapy.

By 2.1 years of age, GC therapy was discontinued; sJIA-targeted therapy was also subsequently discontinued (at 3.5 years of age). During the follow-up visits, she showed marked improvement in her height velocity (Fig. 1A), a persistently normal LS aBMD Z-score trajectory (Fig. 1B), and early signs of vertebral body reshaping but with superior endplate depression at T12 and L1 (Fig. 1C). At 5 years of age, there was ongoing normalization of T12 and L1 vertebral heights in the absence of bone protection (bisphosphonate) therapy, but persistent irregularity and depression of the T12 and L1 superior endplates. This finding was consistent with residual fracture healing plus intervertebral disk herniation (similar to the appearance of Schmorl's nodes) (Fig. 1C). At 10 years of age, she remained in clinical remission, continued off sJIA-targeted therapy, demonstrated a normal LS aBMD Z-score for height (+0.2, height Z-score +0.4) plus near-complete vertebral body reshaping at T12 and L1 (although persistent Schmorl's nodes-appearing superior endplate changes at these levels). She also reported engaging in age-appropriate physical activities without limitations or back pain.

### Patient 2: 5-Year-Old Girl With Juvenile Dermatomyositis

A 5-year-old girl presented for evaluation in the Pediatric Bone Health-Osteology Clinic following a diagnosis of juvenile dermatomyositis (JDM) plus the initiation of high-dose GC therapy 6 months earlier (prednisone 2 mg/kg/day, 202 mg/m^2^/day in HCE). At the time of referral, she was also receiving IV immunoglobulin, methotrexate, and had started a GC wean following favorable clinical evolution on combinatorial therapy. At the time of the bone health assessment, she was on prednisone 0.6 mg/kg/day (75 mg/m^2^/day in HCE) and reported minimal, midthoracic back pain not interfering with day to day activities, absence of nonvertebral fractures, and normal mobility.

**Figure 2.**
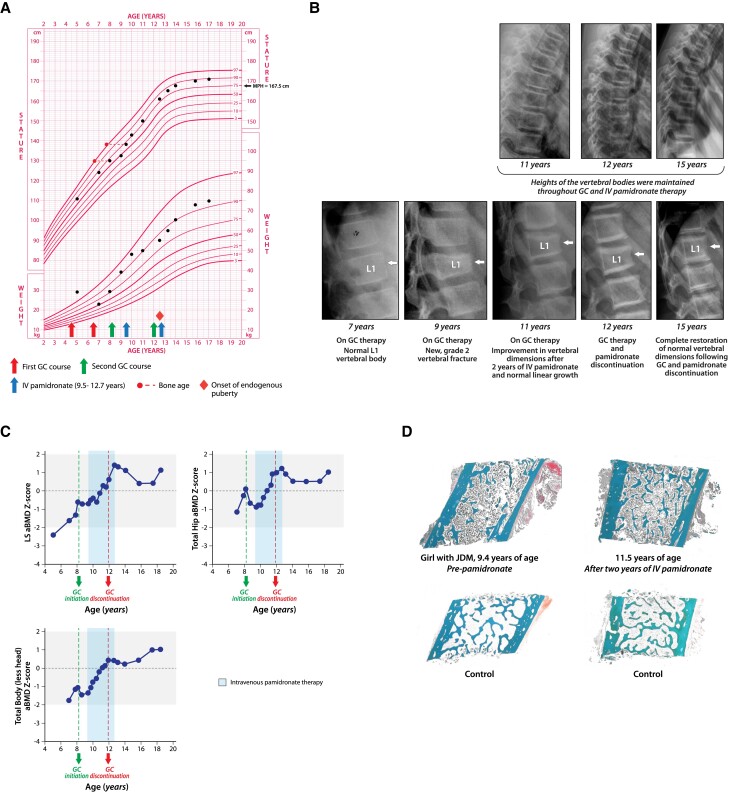
Patient 2. (A-D) The skeletal health of a girl with GC-treated juvenile dermatomyositis who received 2 courses of daily, supraphysiological GC therapy for the treatment of her rheumatic condition; the second course was associated with vertebral fractures and an ongoing need for GC treatment, prompting IV bisphosphonate therapy. (A) Weight loss during the first GC wean, growth deceleration with the second GC course, and attainment of a normal adult height in the years following her GC wean. These observations were consistent with absence of a permanent GC effect on height plus well-controlled underlying disease during the critical growing years. (B) The patient's vertebral fracture trajectory, including vertebral body reshaping plus densification of the vertebral endplates on pamidronate and ongoing vertebral body reshaping post-GC and postpamidronate discontinuation. The thoracic spine is shown at the top (where available) and lumbar spine at the bottom. Her vertebral fracture evolution on these spine x-rays was as follows: normal L1 vertebral body at 7 years of age (thoracic and lumbar SDI = 0). At 9 years of age, she developed an incident (new) Genant Grade 2 vertebral fracture at L1 (SDI = 2); at 11 and 12 years, she demonstrated L1 partial reshaping (SDI = 1) followed by complete vertebral body re-shaping at 15 years of age (SDI = 0). (C) The patient's multisite BMD parameters by DXA, highlighting the supra-physiological aBMD Z-score trajectories at all 3 sites (LS, total hip, and TBLH) on IV pamidronate, while also receiving GC therapy. The uniform decline in BMD parameters postpamidronate cessation was expected (with values remaining well within the normal range), as the patient returned to her normal “endogenous” BMD set-point following bisphosphonate discontinuation. (D) The patient's transiliac bone sample taken for bone histomorphometry prepamidronate and 2 years postpamidronate, highlighting the main effects of IV bisphosphonate therapy on bone tissue in children, increases in cortical width and in trabecular number (both growth mediated). This outcome is caused by the antiresorptive effect of bisphosphonate therapy on the endocortical surface combined with the uncoupled, innate periosteal apposition that occurs during growth on the periosteal surface. The observed bone tissue effects on IV pamidronate underscore the importance of treating osteoporosis during the growing years, a period that is exquisitely sensitive to the bone density- and structure-enhancing effects of bisphosphonate therapy. Abbreviations: a, areal; BMD, bone mineral density; DXA, dual-energy x-ray absorptiometry; GC, glucocorticoid (s); IV, intravenous; JDM, juvenile dermatomyositis; L, lumbar; LS, lumbar spine; MPH, midparental height; SDI, Spinal Deformity Index; TBLH, total body less head.

On physical examination, she appeared Cushingoid, with linear growth arrest noted since GC initiation (Fig. 2A). Her spine was straight and there was no pain on palpation over the posterior spinous processes. Her laboratory evaluation showed an adequate 25OHD concentration on vitamin D_3_ 400 IU daily. A lateral spine radiograph demonstrated Genant Grade 1 (mild) anterior wedging of T6 and T7 vertebral bodies (SDI = 2, x-ray not available), and her LS aBMD Z-score was −2.4 (low for her height Z-score of +0.4, Fig. 2C). A decision was made *not* to initiate bone protection therapy at that time, given her potential for recovery that included young age (prepubertal), the GC weaning process, and only mild vertebral collapse with minimal back pain that did not interfere with quality of life.

At her follow-up visits, she continued to demonstrate increases in height velocity and in LS aBMD Z-scores consistent with GC weaning; GC therapy was then discontinued at 6.5 years of age. At 8.1 years of age, she experienced a JDM relapse and restarted supraphysiologic GC therapy (prednisone 0.5 mg/kg/day, 60 mg/m^2^/day in HCE) along with cyclosporine. A repeat lateral spine x-ray showed failure of reshaping of the previous anterior wedge fractures at T6 and T7 (x-ray not available) plus a new vertebral fracture at L1 (Genant Grade 2; total spine SDI = 4, Fig. 2B). A transiliac bone biopsy (a technique restricted to highly specialized centers) was most notable for a profoundly reduced bone formation rate/bone surface at 19% of the healthy average, Fig. 2D, left panel). Given the progressive vertebral collapse and reinitiation of GC therapy, she was started on IV pamidronate at the age of 9.5 years (Fig. 2B). Her multisite aBMD Z-score parameters prepamidronate were low for her height Z-score: LS −0.7, total hip −0.7, and total body less head (TBLH) −1.5; height Z-score +0.6 (Fig. 2C).

After 3 years of IV pamidronate, she showed improvement in her vertebral dimensions (Fig. 2B), increases in LS, Total Hip, and TBLH aBMD Z-scores (Fig. 2C), complete resolution of her back pain, and normal height velocity (Fig. 2A). At that time, her JDM was well-controlled on mycophenolate mofetil and plaquenil; therefore, her GC therapy was weaned to discontinuation at the age of 12 years, followed by discontinuation of pamidronate at the age of 12.7 years. In the years after GC and pamidronate discontinuation, she achieved further reshaping of vertebral bodies (Fig. 2B), ongoing increases in her multisite aBMD Z-score parameters (LS BMD Z-score +1.3 [Δ + 2.0 compared with prepamidronate], total hip +0.9 [Δ + 1.6], and TBLH +0.3 [Δ + 1.8]; height Z-score at the time was +0.9 (Fig. 2C). In addition, she demonstrated an increase in her transiliac cortical thickness and trabecular number (Fig. 2D, right panel). She was followed until 18 years of age; during this time, she required occasional, short bursts of supraphysiologic pulse GC therapy for flare-ups and underwent menarche at 12.5 years of age. At the last follow-up, she had complete restoration of normal vertebral dimensions following GC and pamidronate discontinuation (first evident at 15 years of age, SDI = 0, Fig. 2B) and an appropriate LS aBMD Z-score for age, sex, and height (Fig. 2C, height Z-score +1.2).

### Patient 3: 6.9-Year-Old Boy With Duchenne Muscular Dystrophy

A 6.9-year-old boy was seen in the Pediatric Bone Health-Osteology Clinic for a routine skeletal health assessment in the context of GC-treated DMD. On history, he was diagnosed with DMD (deletion of *DMD* exons 48-51) at the age of 3.5 years. He was started on deflazacort 0.9 mg/kg/day (60 mg/m^2^/day in HCE) (10) at 5.9 years of age. At first assessment, he had no back pain nor history of fractures. He was mobile, including climbing the stairs, although slower relative to peers. He was on vitamin D_3_ 800 IU daily and had been for 2 years. On examination, his height Z-score was −1.1, and weight Z-score −0.5. There was no evidence of Cushingoid features, his spine was straight, and there was no pain on palpation over the posterior spinous processes. His multisite aBMD Z-scores by DXA showed the following: LS −1.8, total hip −1.8, and TBLH −2.1 (all low for height). His lateral spine x-ray showed no evidence of vertebral fractures.

**Figure 3.**
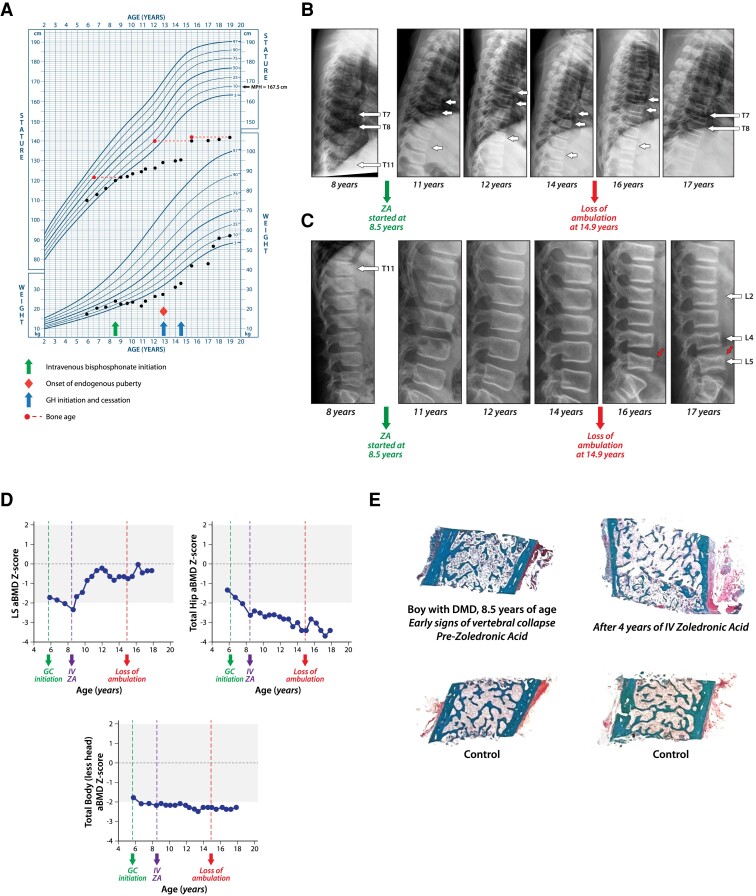
Patient 3. (A-E) The skeletal health trajectory of a boy with DMD who started daily, supraphysiological GC therapy at 5.9 years of age and IV pamidronate at 8 years for early signs of vertebral collapse. In contrast to patients 1 and 2, this patient had persistent and permanent threats to bone health, including long-term GC therapy plus a progressive myopathy. These “disease context” features have led to guidelines that support initiating bone protection therapy at early, rather than late, signs of bone fragility in individuals with DMD. (A) describes the profound growth arrest of GC-treated DMD, including lack of response to rhGH. The slight improvement in linear growth after 14 years of age was attributed to progression through endogenous puberty. Spontaneous puberty in the daily GC-treated DMD setting is unusual; the need for pubertal induction is more typical. (B and C) The patient's vertebral fracture trajectories. (B) Thoracic spine. Demonstrates minimal anterior wedging at T11 that did not quite meet fracture criteria at 8 and 11 years of age (defined as >20% loss of vertebral height ratio); however, given the natural history of progressive vertebral collapse in GC-treated DMD combined with a significant decline in total hip aBMD Z-score, bone protection therapy (IV ZA) was initiated at 8 years of age. At 12 years of age, he demonstrated an incident (new) Genant Grade 1 vertebral fracture at T7. At 14, 16, and 17 years of age, the Grade 1 vertebral fracture at T7 was stable, with evidence for a global decrease in height at T8 that did not quite meet fracture criteria. (C) Lumbar spine. Shows normal lumbar vertebrae at 8 and 11 years of age, followed by a mild decrease in height at L3 (at 12 years of age) that did not meet the fracture criteria. At 16 years of age, there was a new Genant Grade 2 vertebral fracture at L5 with buckling of the anterior cortex at the superior endplate (diagonal arrow) and a new Genant Grade 1 vertebral fracture at L4. There was also a Grade 1 monoconcave fracture at L2 with loss of endplate parallelism. At 17 years of age, his spine x-ray was stable relative to the previous. At 17 years of age, his vertebral deformities remained stable (while continuing IV bisphosphonate therapy as he transitioned to adult care). (D) The multisite aBMD Z-score trajectories on IV ZA therapy, showing favorable evolution at the spine, progressive decline despite bisphosphonate therapy at the hip, and stabilization for the TBLH measurement. (E) The bone structure on transiliac bone biopsy in GC-treated DMD treated with IV bisphosphonate therapy. Unlike in patient 2 (Fig. 2D), the dystrophinopathy plus GC-induced growth impairment in patient 3 prevented the typical growth-mediated increase in cortical thickness and trabecular number on potent antiresorptive therapy. Nevertheless, it was anticipated that the degree of osteoporosis on transiliac bone biopsy would have been more severe in the absence of treatment. Abbreviations: a, areal; BMD, bone mineral density; DMD, Duchenne muscular dystrophy; GC, glucocorticoid; GH, growth hormone; IV, intravenous; rhGH, recombinant human growth hormone; L, lumbar; MPH, midparental height; SDI, Spinal Deformity Index; T, thoracic; TBLH, total body less head; ZA, zoledronic acid.

At his 1-year follow-up visit (8 years of age), he had an intermittent history of back pain but lack of nonvertebral fractures. He did not develop significant weight gain on deflazacort, but he did have a decline in his linear height velocity (Fig. 3A) with mild Cushingoid features. There was mild tenderness on palpation over the posterior spinous processes in the midthoracic region, and he was pre-pubertal. Serum c-telopeptide of type I collagen and bone-specific alkaline phosphatase were below average at this time (Z-scores for both −1.5) and remained low thereafter. He had significant BA delay for a chronological age of 9 years, though phalanges were less delayed (6-7 years) than carpals (5 years). Biochemical parameters of bone and mineral ion metabolism were unremarkable, including a serum 25OHD concentration. Lateral spine x-ray showed minimal anterior wedging at T11 that did not quite meet fracture criteria but that was also not consistent with anterior physiological rounding (SDI = 0) (Fig. 3B and 3C) and LS aBMD Z-score declined to −2.3 (Δ −0.5 from baseline at 6.7 years of age, Fig. 3D). Given minimal changes at T11 on his lateral spine x-ray (accompanied by declines in LS aBMD Z-scores) in the context of persistent risk factors and a disease setting known to be at high risk for fragility fractures, he was started on IV zoledronic acid (ZA) at 8.5 years of age (annual dose 0.1 mg/kg/year divided 6 monthly), following a transiliac bone biopsy (Fig. 3E).

His back pain resolved completely post-ZA. In view of his linear growth delay, he underwent endocrine testing that revealed normal thyroid function and insulin-like growth factor-1 concentrations; however, he failed a clonidine and arginine growth hormone stimulation test (peak growth hormone 2.2 µg/L at the age of 10.3 years, N > 10). He started recombinant human growth hormone (rhGH) at 13 years of age (0.03 mg/kg/dose, 6 days per week); this was tolerated uneventfully but was ultimately discontinued at the patient's request after 1.5 years due to lack of significant improvement in height velocity despite prescription adherence (Fig. 3A). He had spontaneous puberty at the age of 13 years and complete loss of ambulation at 14.9 years.

At 10.8 years of age, his IV ZA dose was decreased from 0.1 mg/kg/year to 0.05 mg/kg/year, given his stable vertebral fracture status and normal LS aBMD Z-score; he remained on this dose throughout his pediatric years. At his last visit (17.8 years of age), he continued to receive IV ZA therapy, with marked improvement in his LS aBMD Z-score (−0.3 at the most recent visit; Δ +2.0 compared with pretreatment), whereas his total hip aBMD Z-score progressively declined (−3.5 at most recent visit; Δ −0.8). His TBLH aBMD Z-score was stable, and his height Z-score remained low at −4.4 (Fig. 3A and 3D**)**. Despite significant improvements in LS aBMD Z-scores on IV ZA throughout childhood (Fig. 3D, also evident on lateral spine x-ray as densification of the vertebral endplates), his x-ray showed mild, progressive vertebral height loss, particularly in the lumbar region (all asymptomatic, Fig. 3B and 3C). Figure 3E shows the patient's response to bisphosphonate therapy on transiliac bone biopsy. Unlike in patient 2 (Fig. 2D), the dystrophinopathy plus GC-induced growth impairment in patient 3 prevented the typical growth-mediated increase in cortical thickness and trabecular number on potent antiresorptive therapy. Nevertheless, it was anticipated that the degree of osteoporosis on transiliac bone biopsy would have been more severe in the absence of treatment. At the time of discharge to adult care at 17.8 years of age, his BA was close to 16 years at the phalanges and 12.5 years at the carpals. Therefore, he was recommended to continue pediatric IV ZA dosing (0.05 mg/kg divided every 6 months; maximum dose 4 mg) and to transition to annual adult dosing once epiphyses had fused (4 mg per dose). Upon transition, his pediatric to adult transition physicians discussed that he would continue bone protection for as long as he was on GC therapy or longer if there was back pain or new fractures (provided the benefit:risk profile for doing so remained favorable).

## Background

Most of the underlying diseases for which GC are prescribed carry an increased risk of skeletal fragility, particularly inflammatory disorders and hematological malignancies because of the adverse effect of disease-related cytokines on skeletal metabolism (eg, interleukin 1 and 6, tumor necrosis factor-alpha) (11), and neuromuscular disorders such as DMD. Even beyond the skeletal offloading of DMD, the aberrant muscle–bone interactions in this condition that involve muscle-derived myokines, bone-derived osteokines, and shared cytokines catalyze common signaling pathways to incite muscle fibrosis, inflammation and bone loss (12).

**Figure 4.**
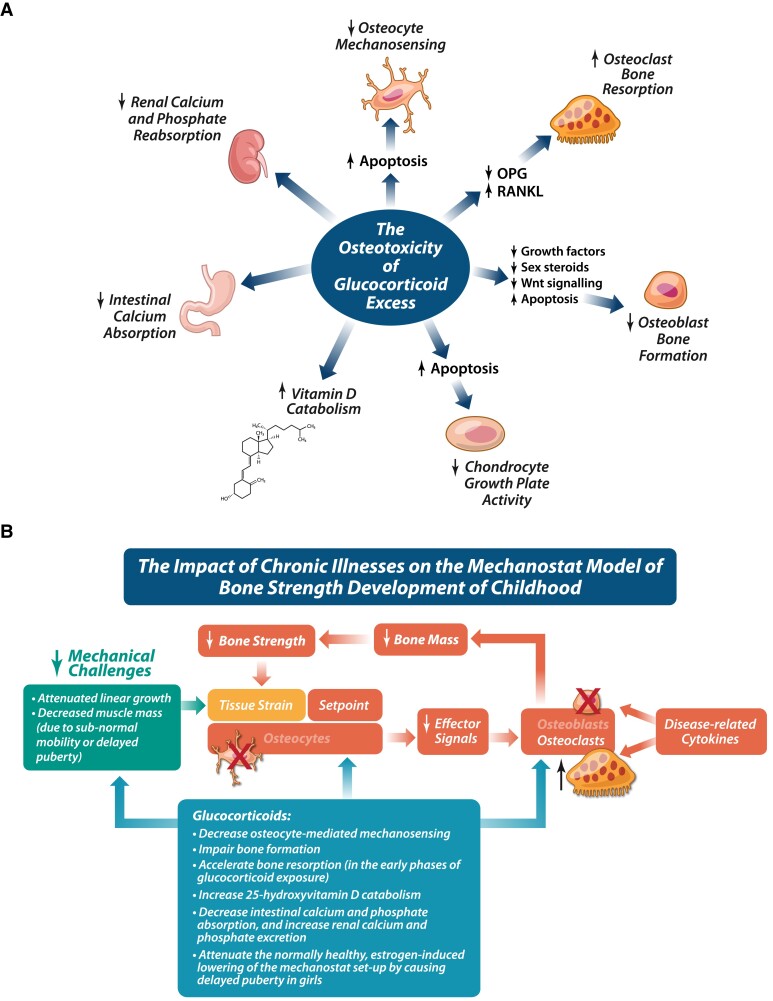
(A) The deleterious effect of GC therapy on bone and growth plate metabolism in childhood. (B) The most important functional consequences of GC-induced bone and growth plate toxicity. Abbreviations: GC, glucocorticoid; OPG, osteoprotegerin; RANKL, receptor activator of nuclear factor kappa-Β ligand.

In addition to disease-related factors, GCs have both direct and indirect adverse effects on the growth plate and developing skeleton, as recently reviewed in detail (13, 14), and outlined in Fig. 4A. As shown in Fig. 4B, GCs wreak havoc on the mechanostat model of bone strength development by interfering at virtually every stage of the cascade (13, 15, 16); therefore, it is not surprising that GCs are potent disruptors of bone strength.

**Figure 5.**
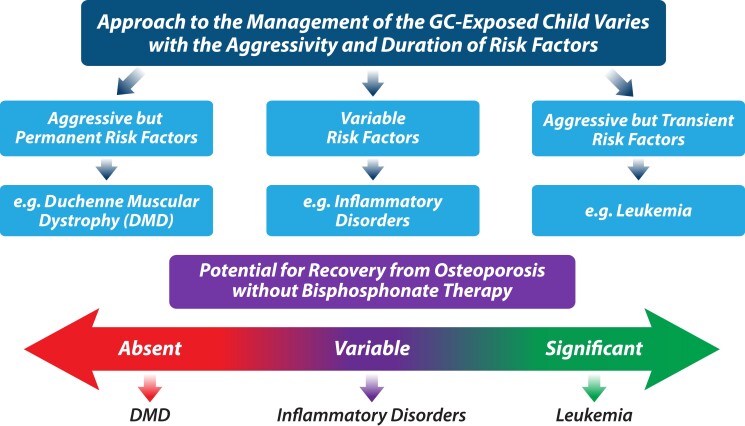
An overall, thematic approach when considering the management of skeletal health in GC-treated children. The approach to GIO management in children is intimately linked to the known fracture risk in various disease contexts and whether risk factors are transient, variable or persistent. These profiles, in turn, determine whether a child is able to recover spontaneously from bone health threats (without osteoporosis therapy), or require bone protection (ie, bisphosphonate therapy). Abbreviations: DMD, Duchenne muscular dystrophy; GC, glucocorticoid; GIO, glucocorticoid-induced osteoporosis.

To unravel the myriad GC effects on the developing skeleton from a practical perspective, natural history studies have taught us to categorize GC-treated children into 3 overarching groups (Fig. 5): those with aggressive but more limited GC exposure (eg, children with leukemia), those with variable GC exposure (eg, children with GC-treated inflammatory disorders and nephrotic syndrome), and those with aggressive and long-term (even permanent) GC exposure (eg, GC-treated DMD). This categorization tunes the practitioner to a pivotal consideration that distinguishes pediatric from adult GIO management in a fundamental way: whether a child is likely to fully recover from GC-induced osteotoxicity in the absence of bone-targeted therapy (including reclamation of normal vertebral dimensions following vertebral fractures). The “potential for medication-unassisted recovery from GIO” is indeed central to the overall approach in children, as discussed in the ensuing sections.

## Monitoring and Diagnosis

One of the most important lessons arising from the STOPP study was that vertebral fractures are more frequent in GC-treated children than nonvertebral fractures, underscoring the vulnerability of the trabeculae-rich spine to the adverse effects of GCs. Pediatric vertebral fractures are extremely rare in the absence of trauma (17), but studies have shown that in the 6 years following GC initiation, with vertebral fractures occurred in one-third of children with leukemia and 16% of children with rheumatic conditions (4, 7) and up to 75% of boys with GC-treated DMD (18). By demonstrating that vertebral fractures defined according to the modified Genant semiquantitative method (19, 20) associated with clinically relevant variables including LS aBMD Z-scores, back pain, second metacarpal percent cortical area, and an increased risk of future fractures (1, 4, 6, 21), the STOPP Consortium validated that >20% loss in vertebral height ratio is an acceptable definition of a vertebral fracture in children.

**Figure 6.**
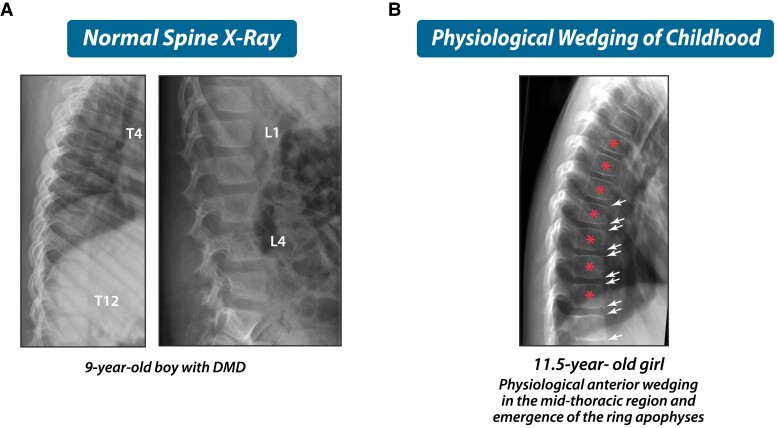
(A, B) Examples of normal spine x-rays in children, including “anterior physiological rounding” in the midthoracic region (stars, B). Physiological wedging is apparent in younger children due to lack of, or emerging, ossification of the ring apophyses. This “normal anterior physiological rounding” is distinguished from vertebral fractures in 2 ways. First, the anterior:posterior height ratio will not meet fracture grade (ie, will not reach >20% loss in anterior:posterior height ratio). In addition, there is a slight “posterior bulge” of the vertebral body in physiological rounding. The arrows show early signs of ring apophysis ossification in this child; once ossification of the ring aphophyses is complete, the vertebral bodes take on the more classic “square-like” shape of the mature spine. Abbreviation: DMD, Duchenne muscular dystrophy.

**Figure 7.**
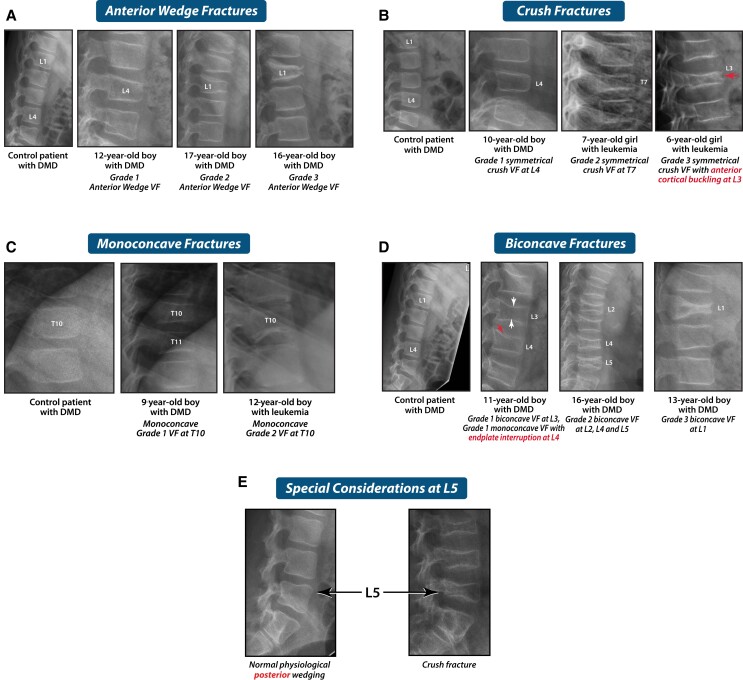
(A-E) Examples of vertebral fractures in children with severity grading according to the Genant semiquantitative method (see text). These include anterior wedge fractures (the most common deformity (A)), crush fractures (B), monoconcave and biconcave fractures (C and D). L5 has a unique posterior wedge shape that is not part of the formal Genant semiquantitative assessment, but can nevertheless undergo fracture (E), particularly in patients with hyperlordosis as in DMD. In addition, qualitative signs of vertebral fractures may be evident, including loss of endplate parallelism (shown throughout these images, a phenomenon which occurs when there is asymmetrical vertebral height loss within and relative to adjacent vertebral bodies), as well as anterior cortical buckling and endplate interruption. Abbreviations: DMD, Duchenne muscular dystrophy; VF, vertebral fracture; L, Lumbar; T, thoracic.

The most compelling observation that validated the use of the Genant semiquantitative method in children arose from the longitudinal study of children with leukemia, where Genant-defined vertebral fractures at diagnosis were a strong predictor of new vertebral *and* long bone fractures over the ensuing 5 years (4). Lateral spine imaging by conventional radiography or DXA-based vertebral fracture assessment (22) should be evaluated by a health care professional with trained expertise, including the ability to distinguish vertebral fractures from normal pediatric variants (23). Figure 6A benchmarks images in this review to a normal spine radiograph in a GC-treated child, and Fig. 6B provides an example of normal anterior physiological rounding in the midthoracic region; such anterior rounding is characteristic of the immature spine and must not be mistaken for a Grade 1 fracture. Figure 7A-7E shows signs of vertebral fractures according to the Genant semiquantitative method; in equivocal cases, qualitative signs of vertebral fractures may aid the clinician in the diagnosis (endplate interruption, anterior cortical buckling, and loss of endplate parallelism). Genant-defined severity grades correspond to the degree of vertebral height ratio loss as follows: Grade 1 (mild) >20 to ≤25%, Grade 2 (moderate) >25% to ≤40%, and Grade 3 (severe) >40% (19, 20).

Endplate interruption and anterior cortical buckling are usually associated with more than 20% reduction in vertebral height ratio. Loss of endplate parallelism can be apparent in vertebral bodies which fall short of reaching fracture grade but are nevertheless on the pathway to fracture, and with anterior physiological rounding. Once a vertebral body reaches fracture grade, loss of endplate parallelism is almost universal (the exception being symmetrical crush fractures). Symmetrical crush fractures are at greatest risk of going undetected because they retain a relatively normal overall vertebral shape; this is especially true when there are multiple adjacent crush fractures. Such fractures are assessed by semiquantitative comparison of the posterior height of the vertebral body in question to the posterior height of the vertebral body above (to determine if there is >20% loss in height ratio); widening of the intervertebral spaces may also provide visual clues to the diagnosis of symmetrical crush fractures.

Even a single, low-trauma long bone fracture can be a major osteoporotic event in children with GC-treated disorders. Although forearm fractures are extremely common in childhood, the clinical context surrounding the fracture (low or high trauma), plus the GC-treated child's clinical profile (height, body mass index, puberty, BMD trajectories, GC dose and duration, presence or absence of VF, Cushingoid appearance, and disease activity) usually provide sufficient information to aid the physician in assessing the fracture's clinical significance. Comminuted fractures, and those with atypical displacement, are also significant, especially in the absence of trauma. Lower extremity fractures are particularly concerning in GC-treated DMD due to their association with permanent, premature loss of ambulation (8), along with unexpected mortality due to fat embolism syndrome (24).

One of the questions with which clinicians often struggle is when to initiate bone health monitoring in a GC-treated, or previously GC-treated, child. Vertebral fractures have been diagnosed as early as 4 to 6 months following GC initiation in children with GC-treated inflammatory disorders and DMD (21, 25). With this in mind, bone health monitoring, including lateral thoracolumbar spine imaging, should start around the time of GC initiation in long-term, high-dose GC-exposed populations such as DMD (26), and as soon as possible in other diseases where children are anticipated to receive ≥3 months of daily oral or IV GC therapy. The rationale for this guidance is that none of the natural history studies to date in GC-treated children have reported new (incident) vertebral fractures earlier than 3-4 months following GC initiation (when a baseline spine x-ray has been available to discern incident fractures) (21). Furthermore, longitudinal natural history studies have shown that the peak vertebral fracture incidence in childhood leukemia (4) and rheumatic conditions (7) occurs at 12 months following GC initiation, which corresponds with the timing of maximal annual GC exposure. As such, the first year of GC therapy is a critical time to monitor for early signs of bone fragility, based on the premise that this is also likely to be a period of substantial GC exposure.

**Figure 8.**
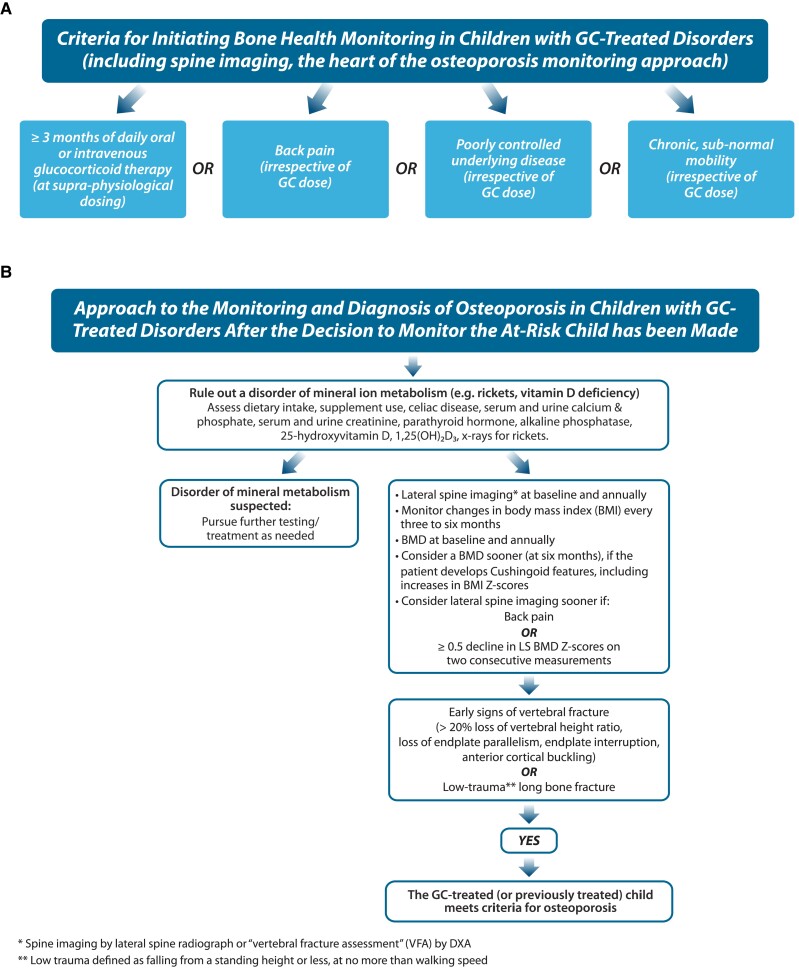
(A) Guidance on the criteria for initiating a bone health evaluation in a GC-treated (or previously GC-treated) child. While daily oral or IV GC therapy for ≥3 or more months at supraphysiological doses is a defining trigger for a bone health evaluation, the presence of back pain, poorly controlled underlying disease, or subnormal mobility are also important considerations in the need for bone health monitoring (irrespective of GC dose, duration or recency of treatment). (B) The overall approach to monitoring and diagnosis of osteoporosis after a decision to monitor an at-risk child has been made. Note that spine imaging (by x-ray or DXA-based “vertebral fracture assessment”) is the fulcrum of the osteoporosis monitoring paradigm in children, given observations that DXA-based BMD Z-scores can be normal in children with overt bone fragility, that BMD Z-scores vary widely depending on the normative data used to generate the Z-scores, and that vertebral fractures (frequently asymptomatic, requiring a proactive approach to their detection in high-risk settings) are a signature manifestation of pGIO. Abbreviations: 1,25(OH)_2_D_3_, 1,25-dihydroxvitamin D_3_; BMD, bone mineral density; BMI, bone mass index; DXA, dual-energy x-ray absorptiometry; GC, glucocorticoid; IV, intravenous; LS, lumbar spine; pGIO, pediatric glucocorticoid-induced osteoporosis.

Figure 8A shows the main factors in deciding whether to initiate bone health monitoring beyond the GC exposure criteria, including back pain, a history of even a single, low-trauma long bone or vertebral fracture, poorly controlled underlying disease, or chronic subnormal mobility (all in the serious acute or chronic illness settings). Figure 8B summarizes the approach to the monitoring and diagnosis of osteoporosis in children with GC-treated disorders after the decision to monitor the at-risk child has been made (26, 27). The heart of the bone health monitoring paradigm is lateral spine imaging at baseline and annually for as long as the patient is GC treated (and potentially longer if there is ongoing, poorly controlled disease, back pain, or subnormal mobility). Baseline and annual BMD parameters (particularly LS and, if the patient is capable, total hip or lateral distal femur BMD) are also suggested given the potential for differential effects of GC therapy and subnormal mobility on the spine vs extremities. Total body (less head) DXA is useful in understanding the GC-treated child's changes in body composition (fat and lean mass). Back pain, increases in body mass index Z-scores, decreases in LS aBMD Z-scores, BA delay, decreases in height velocity, worsening disease activity, and/or Cushingoid features may prompt consideration for an earlier BMD than annually; a drop in LS aBMD Z-score over 2 consecutive measures by ≥0.5 or back pain should also prompt consideration of lateral spine imaging sooner than annually, in order to detect vertebral fractures as close to their first emergence as possible (26).

Over the last decade or so, the pGIO field has moved away from a BMD-centric toward a fracture-focused approach to the diagnosis, for numerous reasons. First and foremost, fractures are the most clinically relevant endpoint, with BMD a surrogate for bone strength. Secondly, BMD can be low due to size artifact (ie, short stature), and Z-scores can decline due to poor linear height velocity, weight loss, or delayed puberty. Furthermore, BMD Z-scores can be greater than −2.0 in GC-treated children with fractures (1, 21, 25), a fact which invalidates the use of a specific threshold to trigger care pathways in the management of pGIO. In addition, BMD Z-scores can vary up to 2.0 SDs depending on the normative data that are used to generate the Z-scores (28), further rendering a BMD threshold–based approach to care pathways impractical. BMD interpretation is therefore most challenging when used as a single cross-sectional measurement. However, as the cases highlight, longitudinal BMD trajectories are highly informative as they typically mirror the child's overall clinical evolution. In routine care, BMD is but one of numerous pieces of information that orients the pediatrician to whether the child's evolution is in a positive or negative direction, whether the child has had a clinically significant fracture, and how the child is responding to bone-targeted treatment.

**Figure 9.**
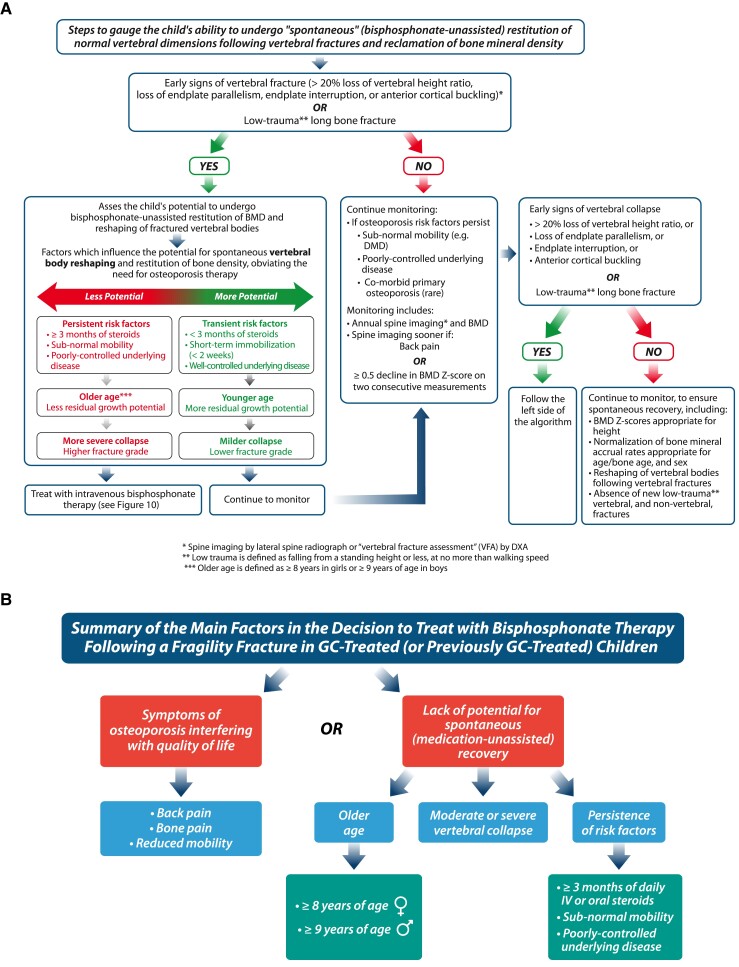
(A, B) Guidance on the initial approach once bone fragility has been identified. The first step (A) is to gauge the child's ability to undergo spontaneous recovery from bone fragility (including vertebral body reshaping and reclamation of a normal BMD for age/bone age, height and sex, thereby obviating the need for bone protection therapy). (B) A summary of the main factors to consider in the decision to treat with osteoporosis therapy. Abbreviations: BMD, bone mineral density; DMD, Duchenne muscular dystrophy; DXA, dual-energy x-ray absorptiometry; GC, glucocorticoid; IV, intravenous; VFA, vertebral fracture assessment.

Once the diagnosis of osteoporosis has been made, the next step is to determine whether the patient has the potential to undergo spontaneous (ie, medication-unassisted) recovery from bone fragility. The juvenile skeleton has tremendous potential to recover from osteoporosis, provided threats to bone health have abated and there is sufficient residual growth potential to permit adequate bone mineral accretion for full recovery. Recovery from osteoporosis does not only involve reclamation of BMD; restoration of normal vertebral dimensions (and thereby total spinal height) is also a key index of recovery (8). A recent study by the STOPP Consortium found in GC-treated children with leukemia, nephrotic syndrome, and rheumatic conditions that patients with higher GC exposure, higher SDI, more severe fractures, and lumbar vertebral fractures were at increased risk for persistent vertebral deformity (8). Figure 9A provides an algorithm which walks the clinicians through the steps involved in understanding a child's potential for medication-unassisted recovery. Figure 9B provides a “quick reference” summary of the main considerations in this pivotal adjudication.

## Prevention and Treatment

Prevention of bone fragility in the GC-treated child starts with conservative measures to optimize bone strength including maintenance of a healthy weight, identifying and treating vitamin D and other nutritional deficiencies, monitoring and treating delayed puberty, fall prevention strategies, and optimization of weight-bearing (within the limits of the underlying disease). Delayed puberty is virtually universal in children with daily, GC-treated DMD. Short stature is a frequent complication of supraphysiological GC dosing that often involves “end-organ resistance” to rhGH therapy due to GC-induced growth plate toxicity; low GH secretory status may also be a factor in GC-induced growth delay. Treatment with rhGH is typically entered into on a case by case basis after careful review of the child's residual growth potential, predicted vs midparental height, anticipated GC prescription, GH secretory status, and psychological orientation to the growth delay. Short stature is often profound in GC-treated DMD at standard, supraphysiological daily doses as shown in patient 3; rhGH is not recommended as standard of care due to insufficient evident about the benefits and risks, but may be considered on a case by case basis (26).

**Figure 10.**
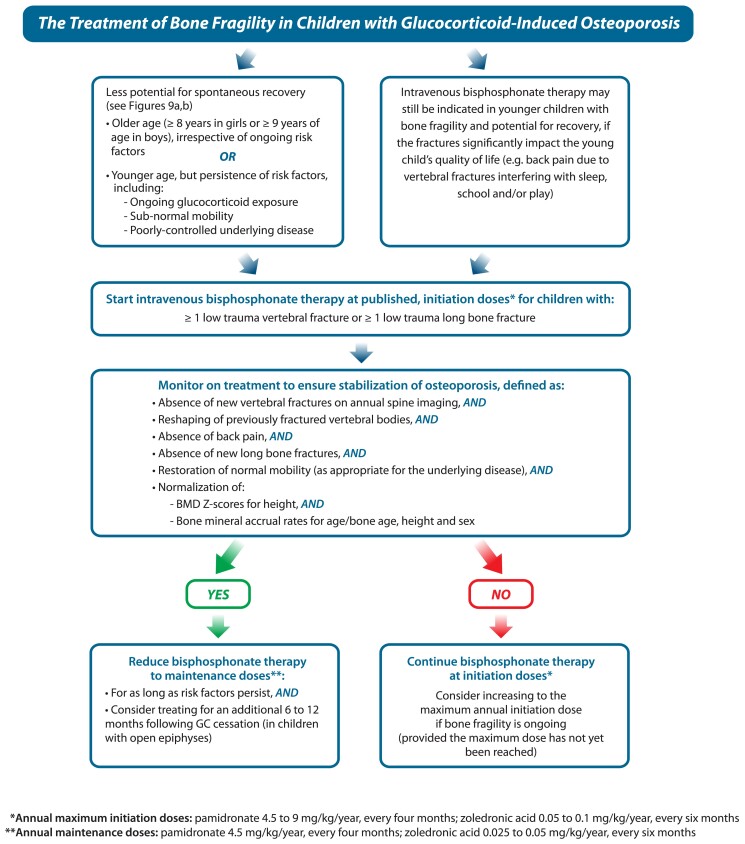
The approach to treatment of pGIO with bisphosphonate therapy once the decision has been made that bone protection therapy is necessary due to lack of potential for medication-unassisted recovery. Abbreviations: BMD, bone mineral density; GC, glucocorticoid; pGIO, pediatric glucocorticoid-induced osteoporosis.

The decision to treat a child with a bisphosphonate or not determines the nature and frequency of follow-up. For children anticipated to recover from osteoporosis, annual follow-up for 1 to 2 years to affirm the anticipated, favorable prognosis is a reasonable approach (including BMD studies, lateral spine imaging if vertebral fractures were part of the osteoporotic phenotype, and a 25OHD concentration). For patients with limited potential for spontaneous recovery, treatment with IV bisphosphonate therapy is the standard of care, Fig. 10). Importantly, a patient's “fitness” for bisphosphonate therapy should be assessed prior to embarking on treatment, including adequate dietary and/or supplemental intake of calcium and vitamin D, euphosphatemia and eucalcemia, 25OHD sufficiency, and adequate renal function. IV ZA is contraindicated in patients with acute renal failure, and dose adjustments to IV therapy are required for patients with estimated glomerular filtration rates less than 60 mL/min/1.73 m^2^. Bisphosphonate-induced hypocalcemia is a well-known phenomenon; however, it is less appreciated that hypophosphatemia is also a frequent consequence postbisphosphonate therapy, particularly in patients with pGIO and other chronic illnesses (29, 30), and may necessitate phosphate supplementation.

The first infusion side effects of IV bisphosphonate therapy can precipitate adrenal insufficiency in patients on chronic GC therapy, even when they are taking daily supraphysiological doses. This is because the GC duration of action on once-daily doses may not be sufficient to cover the entire 24-hour period leading up to the next GC dose (10, 31). Therefore, it is prudent to recommend steroid stress dosing following the first dose of IV bisphosphonates in patients on chronic GC therapy, either prophylactically or upon developing symptoms that would normally prompt such intervention (ie, fever, vomiting) (32, 33). Boys with DMD may experience flu-like symptoms even beyond the first dose and require repeated home management guidelines for steroid stress dosing.

For many years, IV pamidronate was the most frequently used agent in children with osteoporosis following the inaugural, observational study in the late 1990s, which showed improved pain, mobility, and reshaping of vertebral bodies on pamidronate in children with moderate to severe osteogenesis imperfecta (OI) (34). IV ZA has since been introduced, given the advantage that it can be given over a shorter period of time and less frequently (35, 36); ZA is 100 times more potent than pamidronate (37). A randomized study comparing the effect of pamidronate and ZA in OI showed that ZA had similar effects on LS BMD Z-scores and fracture rates over 12 months (35).

Of the oral agents, alendronate and risedronate have been the most extensively studied, with reports confirming that the oral bioavailability of alendronate in GC-treated children is <1%, similar to adults (38, 39). Given the low bioavailability of oral agents, it is not surprising that side effects are also reduced, though at an apparent cost to treatment efficacy.

Despite the formidable challenges in conducting clinical trials in children with osteoporosis, there are now a number of randomized controlled trials of oral and IV bisphosphonates that have been carried out in primary (40-46) and secondary (47-53) osteoporosis of childhood. Collectively, nearly all of the controlled IV *and* oral bisphosphonate therapy trials have shown significant increases in LS BMD Z-scores (both height-adjusted and unadjusted). The 2 routes of administration more clearly distinguish themselves, however, with respect to vertebral body reshaping (in patients with growth potential) (54). Therefore, it is on *these* grounds that the response to IV vs oral bisphosphonate therapy is adjudicated.

Based on observational studies, it is expected that fractured vertebral bodies in OI will undergo reshaping with bisphosphonate therapy (36, 54-56), thereby providing a key index of benefit. The controlled trials to date in growing children with OI which quantified vertebral body height clearly showed increases in those receiving IV bisphosphonate therapy (45, 57, 58), whereas none of the controlled oral bisphosphonate studies in OI where it was measured showed a positive effect on vertebral height (40-42, 59). In fact, vertebral fracture rates were more frequent in patients with OI on risedronate (40), and bone turnover markers increased on average (instead of the expected decrease) on risedronate in the study of children with rheumatic disorders by Rooney et al (48). In a large, randomized trial of daily oral alendronate for moderate and severe pediatric OI (44), there was no effect of alendronate on the cortical width of transiliac specimens. In contrast, this is a key structural index derived from a precise measurement with a known positive response in OI to IV bisphosphonate therapy (60). Overall, these data support the use of IV bisphosphonate therapy first-line in childhood osteoporosis; the increases in BMD on oral agents nevertheless suggest that oral may be a reasonable therapy in situations where IV bisphosphonates are unavailable.

## Back to the Patients

One of the most important concepts that we wish to impart is the expertise that is needed to assess longitudinal spine imaging in children to detect vertebral fractures and to adjudicate changes in vertebral body structure over time according to a standardized approach. The SDI (the sum of the Genant Grades, and therefore a metric of overall spine fracture burden that considers both the number and severity of fractures) is particularly useful in the pediatric setting where static, worsening, and improving vertebral deformity influence management decisions.

Here, we provide examples of quantitative and qualitative signs of vertebral fractures in addition to changes in the SDI over time. These changes run the gamut from spontaneous improvement without bisphosphonate therapy in patient 1 with sJIA, bisphosphonate-assisted vertebral body reshaping while on GC therapy plus ongoing improvement in vertebral dimensions post-GC and postbisphosphonate discontinuation in patient 2 with JDM, and progressive vertebral deformity despite a robust LS aBMD Z-score response and early introduction of bisphosphonate therapy in patient 3 with DMD. The latter is an aggressive form of pGIO, where linear growth is typically affected by the high daily doses and prolonged GC exposure, thereby interfering with growth-mediated vertebral body reshaping.

## Special Considerations in Children at the Extreme Ends of Potential for Recovery From GIO: Leukemia and Duchenne Muscular Dystrophy

**Figure 11.**
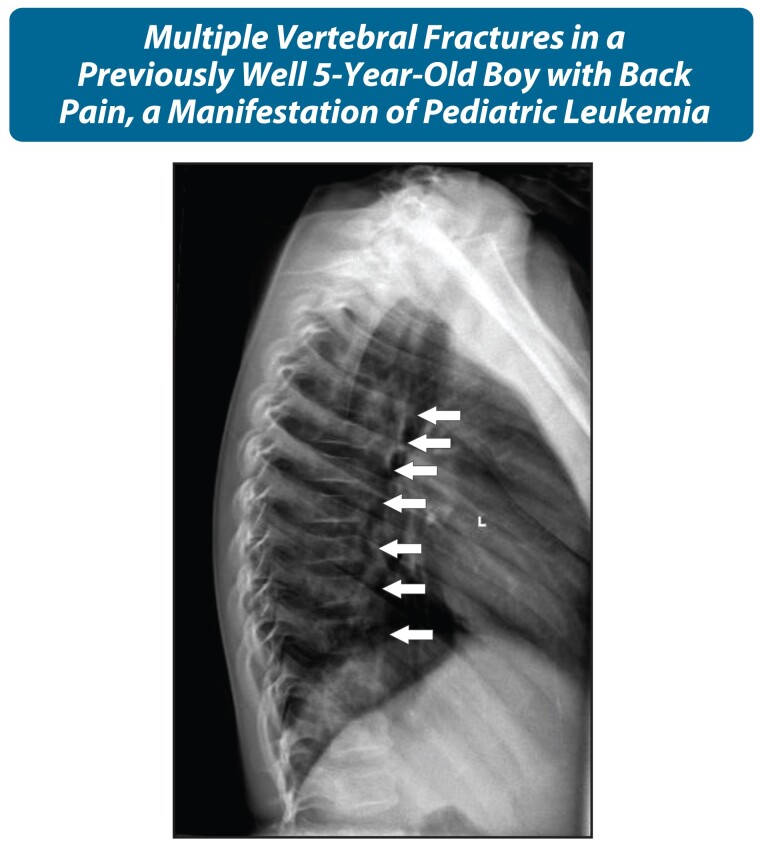
A lateral spine x-ray in a child with painful vertebral fractures due to leukemia, a known manifestation of hematological malignancy that is the diagnosis to rule out urgently. Interestingly, this patient also had a loss of function variant in the *LRP5* gene, which may have exacerbated his risk for low BMD and vertebral fractures. In addition, his CBC was not typical for leukemia at diagnosis (lack of hyperleukocytosis or suppressed marrow activity otherwise), raising the importance of painful vertebral fractures as a hallmark sign of leukemia. Longitudinal studies have shown that nearly 80% of children with vertebral fractures during leukemia therapy will undergo complete vertebral body reshaping without bisphosphonate therapy, with fracture severity (degree of vertebral height loss), fractures in the lumbar region, and residual growth potential negatively influencing the potential for reclamation of normal vertebral dimensions. Abbreviations: BMD, bone mineral density; CBC, complete blood count; *LRP5*, low-density lipoprotein receptor-related protein 5.

Patients at the extreme ends of the potential for recovery from pGIO merit particular consideration. Children with leukemia represent the prototypical example of the potential for vertebral body reshaping in the vast majority. In the longitudinal STOPP study, 16% of children with leukemia had prevalent vertebral fractures at diagnosis (Fig. 11), and one-third of children sustained new vertebral fractures in the ensuing 6 years (most of which occurred in the first 24 months) (4). At the same time, the STOPP Consortium also demonstrated that almost 80% of the children with vertebral fractures underwent complete vertebral body reshaping over 6 years following diagnosis, while the remainder underwent partial (15%) or absent (5%) reshaping (4). The intermittent nature of GC therapy in this setting combined with the typically young age in the majority at diagnosis are proposed to be important drivers of the recovery phenotype. Because of the tremendous drive for recovery from osteoporotic fractures in leukemia, patients with back or bone pain undergo targeted imaging per the usual clinical care response to pain location. Spine imaging and the need for osteoporosis therapy thereafter are then dictated by the child's pain status, BMD Z-score trajectory, potential for recovery from fractures, and functional mobility.

Recent international guidelines recommend carrying out a BMD at the end of leukemia therapy and, if >1.0 SD for age and sex, to repeat the measurement at age 25. For those with lower values, it was recommended to implement the local standard of care due to a lack of evidence otherwise (61). Questions remain about the treatment approach in those with residual low BMD at the end of growth, given data arising from a pediatric leukemia survivor clinic showing an increased frequency of nondigit fractures (62). It is presently unknown whether a course of oral or IV bisphosphonate therapy in those with residual BMD deficits following completion of therapy will reduce the known fracture risk (62, 63); this is an important research question going forward.

**Figure 12.**
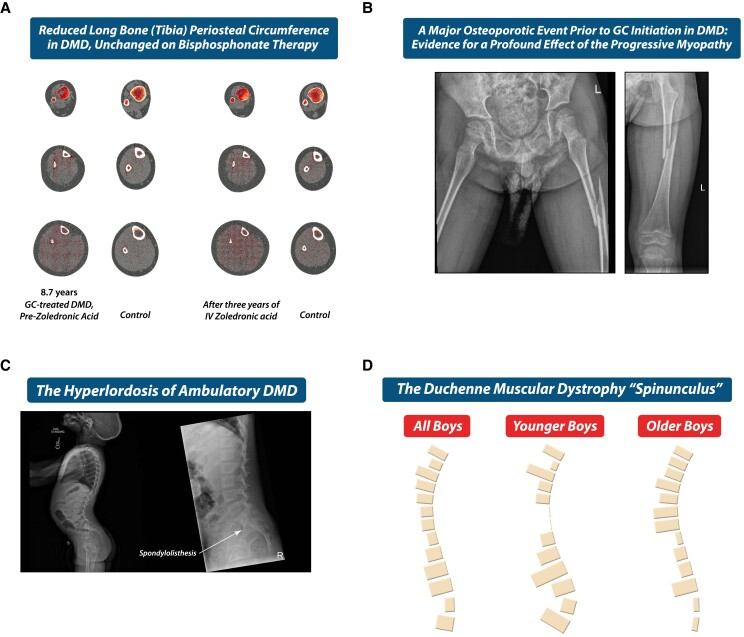
(A-D) highlight special considerations in the forward-looking management of pediatric GC-treated DMD, the most aggressive osteoporosis of childhood with the least potential for recovery. (A) Images by tibia pQCT at the 3% (top row), 38% (middle row), and 66% (bottom row) sites. There is a low trabecular vBMD Z-score at the 3% site prebisphosphonate therapy (−2.2), with improvement post-therapy (+0.2). At the 38% site, there is low periosteal circumference (−1.3) which drops further despite bisphosphonate therapy (−1.6), as expected. The “gracile” (thin) nature of long bones in DMD is a major risk factor for fractures despite potent antiresorptive therapy, since anti-resorptives do not increase bone cross-sectional diameter. Gracile long bones are a well-known feature of osteoporosis due to neuromuscular disorders, a phenomenon that is exacerbated by the growth-attenuating effects of GC therapy. At the 66% site, where muscle is maximal, an increase in muscle-cross-sectional diameter due to fibrosis is evident in the patient with DMD compared with the control. (B) The consequences of long bone structural abnormalities and low BMD in pediatric DMD: a major, low trauma femur fracture prior to GC initiation in a 4-year-old boy. (C) Hyperlordosis (unique to the ambulatory phase of DMD, as loss of ambulation typically results in flattening of the lumbar spine due to hip contractures from prolonged sitting). (D) The DMD “spinunculus” according to age of the patients, which correlates with the stage of the disease. “Spinunculus”, a novel term developed by Dr. Ward's research group, is used to describe the mapping of vertebral fracture frequencies along the length of the spine in a given condition. Note that (C, D) suggest there may be a relationship between spine deformity and the distribution of vertebral fractures in DMD (where a preponderance of lumbar fractures has been observed relative to other GC-treated populations). Together, the observations in Fig. 12 harken the need for a bone protection approach that starts as early as possible in DMD, with an agent capable of not only improving BMD and cortical thickness, but also bone cross-sectional diameter (the latter, which anti-resorptive therapy does not achieve). Effective treatment of the underlying disease with an approach that maximizes muscle strength and minimizes orthopedic spine deformity and fractures as far as possible remain the ultimate goals in this ultra high-risk setting. Abbreviations: BMD, bone mineral density; DMD, Duchenne muscular dystrophy; GC, glucocorticoid (s); IV, intravenous; pQCT, peripheral quantitative computed tomography; v, volumetric.

DMD is at the other end of the spectrum, with an absence of evidence for spontaneous vertebral body reshaping or reclamation of BMD based on current knowledge and experience. Even with early bisphosphonate treatment, bone health deficits persist, including reduced long bone cross-sectional diameter (Fig. 12A and 12B) and progressive vertebral collapse (as shown in patient 3). Tibia peripheral quantitative computed tomography clearly demonstrates in 3 dimensions that small bone cross-sectional area is a defining aspect of the skeletal phenotype in DMD. This structural aberrancy is unchanged on bisphosphonate therapy because antiresorptive treatment does not foster periosteal apposition (a growth–, muscle strength–, and mechanical forces–driven process) (Fig. 12A). The profound effect of the myopathy is shown in Fig. 12B, a 4-year-old with a major femur fracture prior to initiation of GC therapy for DMD.

The progressive vertebral collapse despite normal LS BMD Z-scores for age, sex, and height in IV bisphosphonate therapy in patient 3 is also concerning. Studies have shown that adults with vertebral deformity following vertebral fractures have chronic pain and functional limitations (64); another adult study showed restrictive lung disease in those with vs without vertebral fractures (65). The STOPP Consortium reported reductions in stature among children with leukemia and vertebral fractures (66); whether vertebral fractures are linked in children to restrictive lung disease and excess mortality is unknown. On a positive note, a retrospective study showed that both GC and bisphosphonate therapy were associated with longevity in DMD (67); whether the favorable association between bisphosphonate therapy and longevity was due to relative preservation of vertebral heights, prevention of fat embolism syndrome, or another mechanism remains unclear. We have recently undertaken studies which suggest the hyperlordosis of ambulatory DMD may contribute to the relatively higher rate of lumbar fractures in this setting compared to other GC-treated children (Fig. 12C and 12D) (68); if so, this observation underscores the importance of effective treatment for the underlying myopathy in order to prevent both vertebral body and overall spine deformity.

Current guidelines advocate for continuing bisphosphonate treatment in patients receiving ongoing GC therapy who exhibit moderate to high risk of fracture (69, 70). In DMD, the permanency and high-risk nature of osteoporosis risk factors, including progressive myopathy, loss of ambulation, and high-dose GC therapy, suggest the need for considering long-term bone protection therapy in this setting. However, there are no studies that have formally assessed the risks and benefits of continuing bisphosphonate therapy postepiphyseal fusion among individuals with DMD who initiated therapy in childhood. Further research in this area is needed, given theoretical concern about adverse effects arising from chronic bone turnover suppression in an already low bone turnover state.

Given the tremendous morbidity arising from pGIO in DMD despite the introduction of bisphosphonate therapy at the earliest signs of fractures, efforts are underway to ignite research programs targeting osteoporosis therapy prior to first-ever fractures (including strategies that move beyond monotherapeutic antiresorptive therapy in hopes that osteoanabolic strategies may positively impact not only BMD but also bone structure). One such agent, teriparatide, was studied in 6 individuals with DMD (ranging in age from 13.0 to 22.1 years of age), giving rise to modest increases in PINP and stable BMD trajectories over 1 to 2 years (71). These preliminary data provide proof of principle that further study of osteoanabolic agents is justified in this condition.

Although gene and genetic therapies are now approved in some countries for the treatment of DMD, none of the advanced treatments are robust enough to recommend the removal of GC therapy from the therapeutic regimen (72). This means that GC therapy is here to stay for the foreseeable future in DMD, placing an onus on the pediatric osteology community to work with DMD partners to improve the aggressive pGIO that currently is present in this condition.

Researchers have investigated whether intermittent prednisone (10 days on, 10 days off) are bone- and growth-plate protective in boys with DMD. The short answer is yes (fewer prevalent vertebral fractures) (73), but at a cost to muscle strength relative to daily prednisone (73, 74). Vamorolone, a novel dissociative steroidal drug, has been developed in an effort to “separate the good from the bad” (75-79). In a recent randomized, placebo- and prednisone-controlled clinical trial (NCT03439670) in ambulatory boys with DMD, vamorolone showed comparable efficacy for muscle function relative to daily prednisone, plus improved linear height velocity and bone turnover markers (79). Preliminary, post hoc analyses also suggest vertebral fracture rates may be lower (80); the impact on long bone fractures related to prednisone remains unknown. However, vamorolone also caused dose-dependent adrenal suppression (79), a critical message to convey given the life-threatening nature of this co-morbidity.

## Summary and Conclusions

There are a number of GIO diagnostic and management principles that are similar between children and adults across the lifespan, including the importance of vertebral fractures as a major sign of osteoporosis-related morbidity, and the need for timely identification and treatment of osteoporosis in order to prevent downward health spirals (eg, femur fractures in boys with DMD and in the elderly leading to permanent loss of ambulation). As such, GIO management should be viewed along a continuum, with the stage set in childhood for optimization of bone strength across the lifespan (81). At the same time, there are uniquely pediatric features that require a different approach to clinical care pathways; these are driven by the potential for dramatic densitometric and structural recovery following resolution of risk factors in the presence of residual growth potential. Overall, current evidence affirms the need for proactive approaches in childhood to optimize pediatric outcomes and prevent permanent bone structural damage and BMD deficits later in life.

## Data Availability

Data sharing is not applicable to this article as no original datasets were generated or analyzed for this “Approach to the Patient” manuscript.

## Abbreviations

25OHD: 25-hydroxyvitamin D
BA: bone age
aBMD: areal bone mineral density
DMD: Duchenne muscular dystrophy
DXA: dual-energy x-ray absorptiometry
HCE: hydrocortisone equivalents
GC: glucocorticoid
GIO: glucorticoid-induced osteoporosis
GH: growth hormone
IV: intravenous
JDM: juvenile dermatomyositis
rhGH: recombinant human growth hormone
LS: lumbar spine
OI: osteogenesis imperfecta
pGIO: pediatric glucocorticoid-induced osteoporosis
SDI: Spinal Deformity Index
sJIA: systemic juvenile idiopathic arthritis
STOPP: Canadian STeroid-induced Osteoporosis in the Pediatric Population
TBLH: total body less head
ZA: zoledronic acid
